# Assessing health risks and preparedness strategies in mass-gathering religious events: a retrospective observational study

**DOI:** 10.1186/s12873-025-01293-x

**Published:** 2025-07-21

**Authors:** Huan-Ting Chi, Wei-Kai Liao, Ming-Tai Cheng, Wei-Kuo Chou, Chien-Hao Lin

**Affiliations:** 1 Department of Emergency Medicine, Taichung Veterans General Hospital, Taichung, Taiwan; 2 Department of Emergency Medicine, National Taiwan University Hospital, No.7, Zhongshan S. Rd., Zhongzheng Dist, Taipei City, 100225 Taiwan

**Keywords:** Health risks, Preparedness, Mass-gathering religious events, Heat-related illnesses, Trauma

## Abstract

**Background:**

Mazu pilgrimages are among the largest moving religious events worldwide, involving ceremonies and processions spanning over 300 km in 8–10 days. The massive crowds pose unique public health challenges. This study aimed to assess the health risks and contributing factors of these events to help authorities and local healthcare services better anticipate, prepare for, and mitigate potential health issues during the pilgrimage.

**Methods:**

We conducted a retrospective observational study using patient data from the Emergency Medical Resources Management System of Taiwan’s Ministry of Health and Welfare from 1 January 2018 to 31 October 2024. Records included demographics, means of transport, Taiwan Triage and Acuity Scale (TTAS) level, diagnosis, and disposition for each emergency department (ED) visit related to the two Mazu pilgrimages. Individual ED visits were aggregated into daily counts to estimate daily health impacts. The primary outcome was the daily total number of pilgrimage-related ED visits; secondary outcomes were daily counts for specific diagnoses. Multivariable linear regression was used to examine associations between environmental and event-related factors—including whether the day was the start or end day of the pilgrimage (S-or-E-day), daily walking distance, highest temperature, and relative humidity—and the log-transformed daily ED visit and diagnosis-specific counts.

**Results:**

A total of 1,637 patients visited the ED during Mazu pilgrimages in the study period. Half arrived by ambulance, and 10.8% were triaged as TTAS I/II. Most patients (89.7%) were discharged without admission; only 8.1% were admitted to general wards. Trauma-related diagnoses were the most common (53.7%), with soft tissue injuries (28.1%) and heat emergencies (16.1%) being the most frequent trauma and non-trauma conditions, respectively. The S-or-E-day variable was significantly associated with the daily number of ED visits, trauma, head injuries, orthopaedic injuries, and heat emergencies. Walking distance was linked to ED visits and trauma cases, while highest temperature was linked to heat emergencies.

**Conclusions:**

During Mazu pilgrimages in Taiwan, most patients visiting the ED presented with mild conditions, predominantly minor trauma-related injuries. By monitoring walking distance and temperature, healthcare providers can better anticipate and prepare for cases related to trauma and heat emergencies.

**Clinical trial number:**

Not applicable.

**Supplementary Information:**

The online version contains supplementary material available at 10.1186/s12873-025-01293-x.

## Background

Various mass-gathering events occur throughout the year, including sports games, concerts, New Year parties, and religious activities [1]. The characteristics of these events vary significantly, resulting in different public health challenges. Mass-gathering religious events are unique because they usually involve an extraordinarily large number of participants. One such event is Hajj, typically involving over two million participants annually. Historically, communicable diseases are the most common causes of morbidity and mortality during Hajj [2–4]. In recent years, there have been increasing concerns regarding disruptions in the normal life of patients with chronic diseases, including significant challenges such as severe heat, long walking distances, direct health hazards, and excessive exertion, which further deteriorate health [5–8]. Trauma is also considered a health issue, particularly in the case of overcrowding or stampede [9, 10]. 

**Fig. 1:**
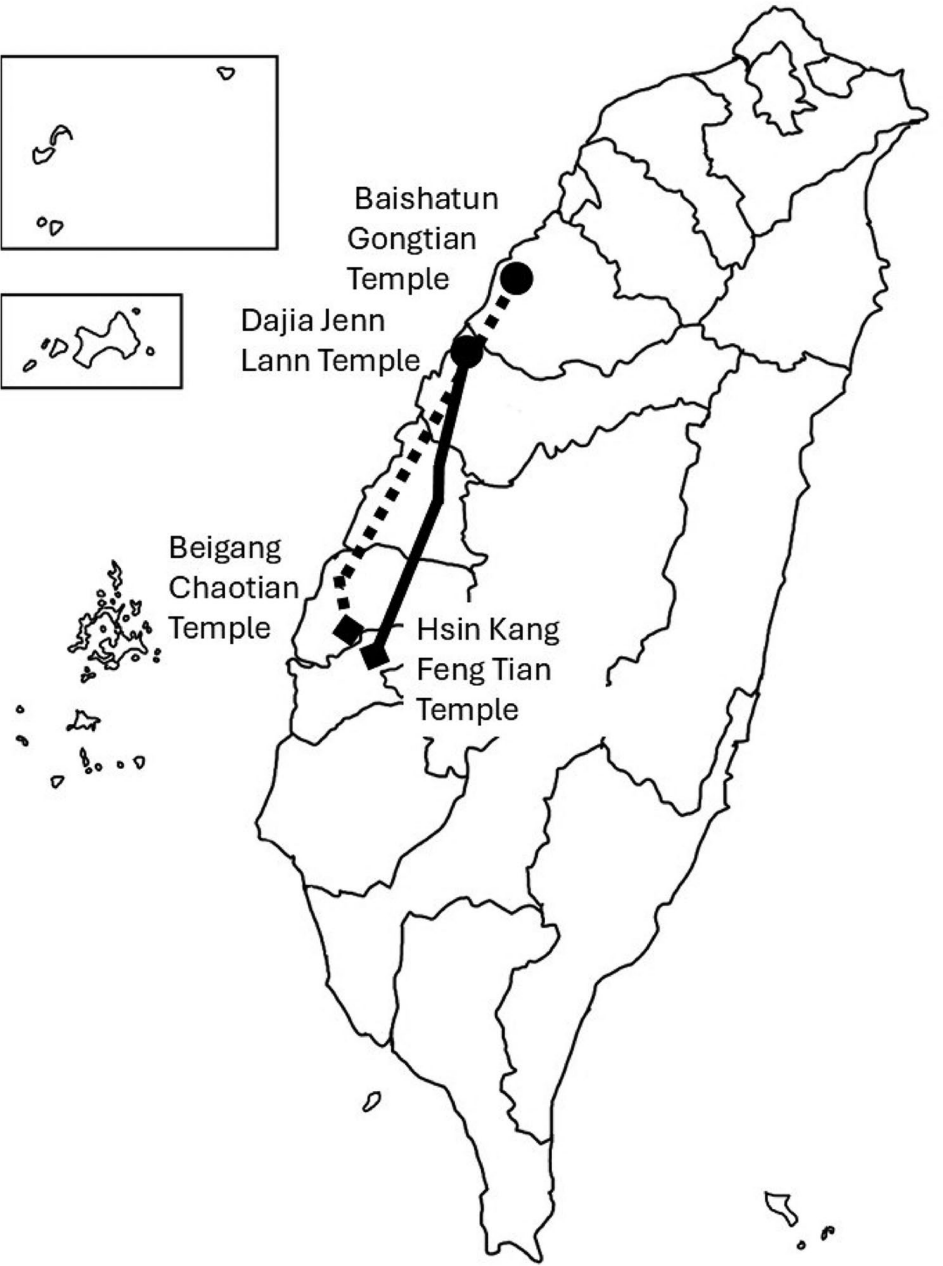
Demonstration of the route of the pilgrimages. The solid line shows that the Dajia Mazu pilgrimage started from the Dajia Jenn Lann Temple and the turning point at Hsin Keng Feng Tian Temple, which covers approximately 333 km. The broken line shows that the Baishatun Mazu pilgrimage started from the Baishatun Gongtian Temple and the turning point at Beigang Chaotian Temple, which is covers approximately 300 km

Mazu pilgrimages, including the Dajia Mazu and Baishatun Mazu, are major religious and cultural events in Taiwan. Held annually in April to celebrate Mazu’s birthday, devotees from all walks of life carry statues of the sea goddess as they visit various temples to express gratitude and seek blessings. These pilgrimages, often likened to the Western Camino, have evolved into large-scale events attracting millions of participants each year. The Dajia Mazu pilgrimage follows a fixed 333-kilometer route from Taichung to Chiayi over 9 days and 8 nights, drawing more than one million participants by 2024. Over 200,000 people attend the opening and closing ceremonies, and tens of thousands walk daily, often from midnight to evening. In contrast, the Baishatun Mazu pilgrimage features a spiritually guided, unpredictable route of around 290 km. Its popularity has surged, from 5,000 participants in 2010 to nearly 180,000 in 2024. (Fig. 1) In recognition of its cultural significance, UNESCO listed Mazu Belief and Customs as Intangible Cultural Heritage in 2009.

Unlike sudden-onset disasters, mass-gathering religious events are typically planned, allowing for some level of medical preparedness. Despite increasing participation, little is known about the real-time health risks and factors contributing to emergency department (ED) visits during these moving religious events. Unlike stationary gatherings, these mobile pilgrimages introduce added complexity in healthcare preparedness due to route uncertainty, prolonged physical exertion, and environmental stressors such as heat and humidity.

This study aimed to assess the health risks of long-distance Mazu pilgrimages and identify environmental and event-related factors—such as walking distance, temperature, humidity, and key ceremonial days—that contribute to increased ED utilization. By analyzing retrospective ED data over a seven-year period, we sought to inform future health planning and response strategies for similar moving mass-gathering events. Findings from this study may help guide public health interventions and optimize resource allocation for large-scale religious events in similar contexts globally.

## Methods

### Study design

We conducted a retrospective observational study by reviewing the medical profiles of patients reported by each receiving hospital during the two Mazu pilgrimages, using data from the Emergency Medical Resources Management System (EMRMS) of the Ministry of Health and Welfare between 1 January 2018 and 31 October 2024. According to the regulations, hospitals that received patients from emergencies or events declared by local or central health authorities must record patient profiles in the system and update the information until the patient is discharged from the ED. The EMRMS is a web-based reporting system designed to collect patient data, including date of visit, sex, age, main diagnoses, Taiwan Triage and Acuity Scale (TTAS) level, disposition after ED visit, and means of transport to the ED during emergencies or disasters. The TTAS is the current triage system used in EDs in Taiwan, and it categorises patients into five levels: level 1 (resuscitation), level 2 (emergency), level 3 (urgent), level 4 (less urgent), and level 5 (non-urgent). (Supplementary Table 1) The Dajia Mazu and Baishatun Mazu pilgrimages have been consistently declared since 2018, with the government prospectively collecting event records for analysis. This study was reviewed by the Research Ethics Committee D of National Taiwan University Hospital and classified as exempt (NTUH-REC No. 202407106 W). Informed consent was waived due to the retrospective nature of the study.

### Dataset and outcome measures

This study analyzed individual ED visit records specifically attributable to participants of the Mazu pilgrimage events. All unrelated ED visits during the same period were excluded. Both pilgrimages followed a typical pattern: each began with an opening ceremony in a relatively confined space near the temple, continued with a long-distance journey to the destination temple, and concluded by returning to the home temple for a closing ceremony similar to the opening.

To estimate the health impact of these events, individual ED visit records were aggregated into daily counts to generate outcome measures for each day of the pilgrimage. The primary outcome measure was the daily total number of pilgrimage-related ED visits. Secondary outcome measures included the daily counts of visits for specific diagnostic categories, means of transport, TTAS level, and disposition categories (e.g., admission or discharge).

Based on the characteristics of the pilgrimage, a binary variable was created to indicate whether an incident occurred on either the start or end day of the pilgrimage (S-or-E-day). These two days primarily involve large-scale ceremonial gatherings and typically attract significantly larger crowds than other days, which may lead to different patterns of healthcare utilization and health effects. The S-or-E-day variable was included as fixed dates in the model.

Environmental and event-related factors included in the analysis were the binary “S-or-E-day” variable, the highest daily temperature, relative humidity, and daily walking distance for both pilgrimages each year (Supplementary Table 2).

### Statistical analysis

For descriptive purposes, we summarized the characteristics of all pilgrimage-related ED visits as total counts and percentages. To illustrate daily variation in healthcare demand, we also calculated the mean and standard deviation of the daily counts for each key variable during the pilgrimage period. Descriptive statistics are presented as percentages, means, standard deviations, and 95% confidence intervals for continuous variables. Spearman’s correlation coefficients were used to analyze the association between the daily total number of ED visits and the daily counts of patients by means of transport, TTAS level, and disposition category, in order to understand the relationship between daily ED volume and patterns of patient transport, triage severity, and hospital admission or discharge. Owing to the non-normal distributions, all variables used in the correlation and regression analyses—including the daily total number of ED visits and the daily counts of each diagnostic category—were log-transformed to reduce skewness, stabilize variance, and approximate normality for parametric analyses. Subsequently, multivariable linear regression analysis was performed to examine the association between environmental factors—including the S-or-E-day variable, highest daily temperature, relative humidity, and daily walking distance—and the log-transformed daily ED visit counts and diagnosis-specific counts. A two-tailed p-value ≤ 0.05 was considered statistically significant. All statistical analyses were performed using JASP, version 0.18.3 (University of Amsterdam, Netherlands).

## Results

A total of 1,637 patients visited the ED during the Dajia Mazu and Baishatun Mazu pilgrimages between 2018 and 2024. The mean age of all patients was 49.1 years (standard deviation [SD] = 11.6), and 49.7% were male. Of the total, half (50.0%, *n* = 819) were transported to the ED by ambulance, and few (10.8%, *n* = 177) were triaged as TTAS level I or II. Regarding patient disposition, 1,469 patients (89.7%) were discharged without admission, 132 (8.1%) were admitted to the general ward, 33 (2.0%) were admitted to the intensive care unit, and three (0.2%) died during the study period (Table 1).

**Table 1:** Descriptive characteristics of all pilgrimage-related emergency department visits in Mazu pilgrimages between 2018 and 2024

Variable	Total *n* (%)	Mean (SD) per day
**Patient characteristics**		
Number of patients	1637	13.2 (10.9)
Age (years)	-	49.1 (11.6)
Male	813 (49.7%)	6.6 (5.9)
**Transportation**		
Ambulance	819 (50.0%)	6.6 (5.6)
Other means	818 (50.0%)	6.6 (6.4)
**Triage**		
TTAS I and II	177 (10.8%)	1.4 (1.6)
TTAS III, IV, V	1460 (89.2%)	11.8 (9.8)
**Disposition**		
Discharge	1469 (89.7%)	11.8 (10.0)
Ward admission	132 (8.1%)	1.1 (1.2)
ICU	33 (2.0%)	0.3 (0.5)
Death	3 (0.2%)	0.0 (0.2)
**Day of the pilgrimage**		
Day 1	336 (20.5%)	24.0 (12.2)
Day 2	159 (9.7%)	11.4 (5.9)
Day 3	120 (7.3%)	8.6 (5.2)
Day 4	87 (5.3%)	6.2 (7.5)
Day 5	131 (8.0%)	9.4 (4.9)
Day 6	119 (7.3%)	8.5 (10.4)
Day 7	197 (12.0%)	14.1 (11.7)
Day 8	259 (15.8%)	18.5 (13.0)
Day 9^a^	216 (13.2%)	19.6 (12.2)
Day 10^b^	13 (0.8%)	13.0 (0.0)

SD: standard deviation; TTAS: Taiwan Triage and Acuity Scale; ICU: intensive care unit

^a^*n*=3 (Year 2020, 2023, 2024)

^b^*n*=1 (Year 2019)

**Fig. 2:**
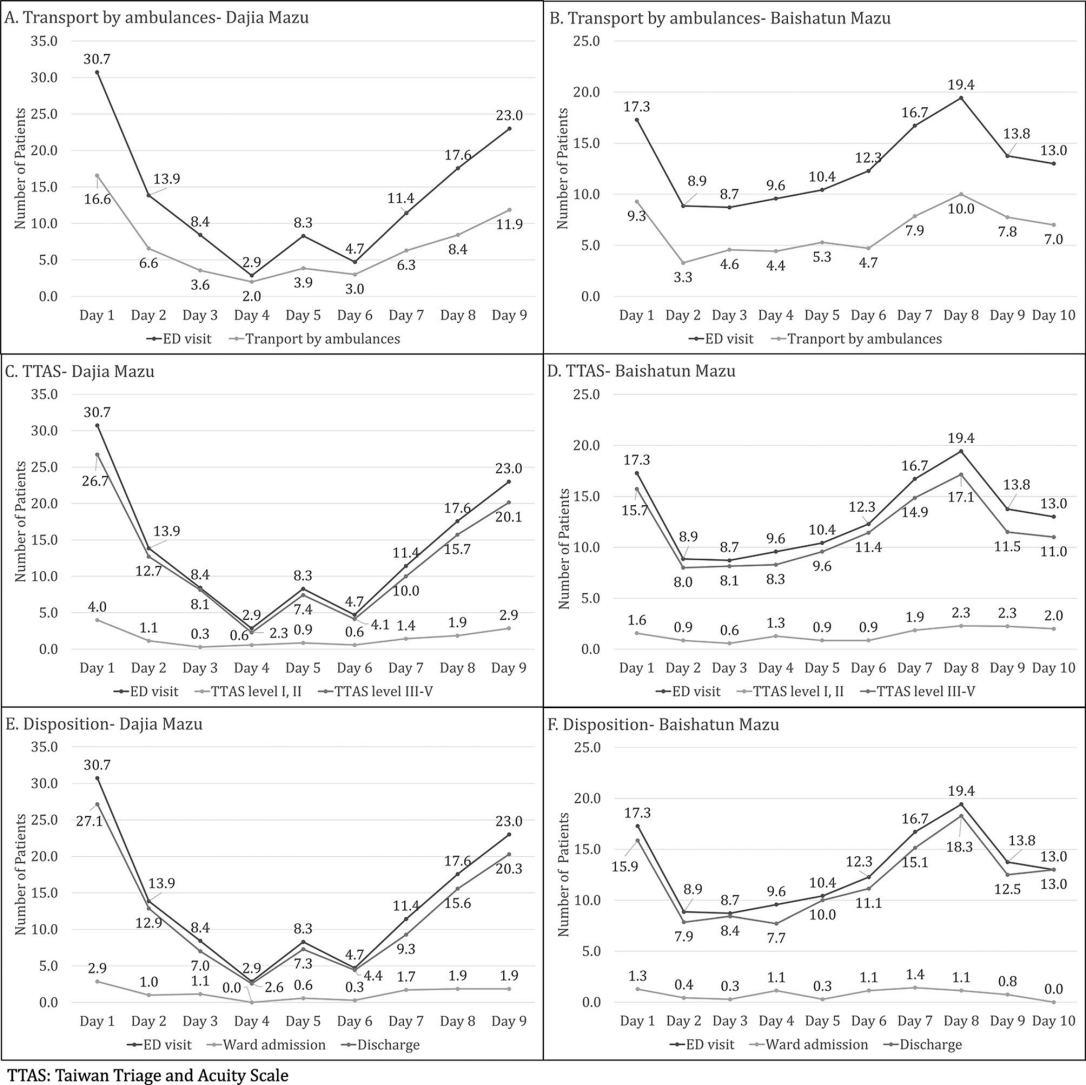
The daily total number of ED visits and the daily counts of patients by means of transport, TTAS level, and disposition category in the Mazu pilgrimages. Panels **A**, **C**, and **E** represent data from the Dajia Mazu pilgrimage, while panels **B**, **D**, and **F** show data from the Baishatun Mazu pilgrimage

The Dajia Mazu pilgrimage typically spanned 9 days, whereas the Baishatun Mazu pilgrimage varied in duration: 8 days long on three occasions, 9 days long three times, and 10 days long once during the study period. The highest number of ED visits was observed on the start day (Day 1) of all events (20.5%, mean = 24.0, SD = 12.2). Figure 2 illustrates the association between the daily total number of ED visits and the daily counts of patients by means of transport, TTAS level, and disposition category, in order to understand the relationship between daily ED volume and patterns of patient transport, triage severity, and hospital admission or discharge. This figure provides an overview of trends and simple correlations, serving as contextual information rather than presenting results from the multivariable regression analysis. Table 2 showed the correlation between the means of transport per day and number of ED visits was strong (Dajia Mazu: *r* = 0.909, *p* < 0.001 vs. Baishatun Mazu: *r* = 0.892, *p* < 0.001) both pilgrimage groups,. Similarly, there was a strong correlation between daily number of patients with TTAS levels III–V and number of ED visits (*r* = 0.989, *p* < 0.001 vs. *r* = 0.994, *p* < 0.001), as well as between daily discharge without admission and number of ED visits (*r* = 0.909, *p* < 0.001 vs. *r* = 0.994, *p* < 0.001).

**Table 2:** Spearman’s correlations between daily ed visits and transport, triage, and disposition counts

Variables	Daija Mazu	*p*-value	Baishatun Mazu	*p*-value
	Spearman’s correlation coefficients		Spearman’s correlation coefficients	
Transport by ambulances	0.909	< 0.001	0.892	< 0.001
TTAS				
Level I-II	0.651	< 0.001	0.77	< 0.001
Level III-V	0.989	< 0.001	0.994	< 0.001
Disposition				
Ward admission	0.684	< 0.001	0.643	< 0.001
Discharge	0.909	< 0.001	0.994	< 0.001

Table 3 summarizes the total number and mean daily number (SD) of patients by diagnosis category during the pilgrimage period. The main diagnoses of most patients (92.6%, *n* = 1517) were reported in the EMRMS. The majority of these patients (53.7%, *n* = 815) had trauma-related diagnoses, with the most common being soft tissue injuries, including abrasions, lacerations, and contusions (28.1%, *n* = 427); followed by head injuries (13.8%, *n* = 209); orthopaedic injuries, including fractures, dislocations, or amputations (8.4%, *n* = 127); and burns (3.1%, *n* = 47).

**Table 3:** The association between the daily total number of ED visits and the daily counts of patients by means of transport, TTAS level, and disposition category in Mazu pilgrimages between 2018 and 2024

Diagnosis category	Total *n* (%)	Mean (SD) per day
**ALL diagnoses**	1517 (100.0)	12.2 (10.9)
**Trauma**	815 (53.7)	6.6 (6.8)
Soft tissue injuries	427 (28.1)	3.4 (3.9)
Head injuries	209 (13.8)	1.7 (2.2)
Orthopaedic injuries	127 (8.4)	1.0 (1.3)
Burn	47 (3.1)	0.4 (0.8)
Torso injuries	4 (0.3)	0.0 (0.2)
**Non-trauma**	702 (46.3)	5.7 (5.2)
Heat emergencies	244 (16.1)	2.0 (2.7)
Skin diseases	55 (3.6)	0.4 (0.8)
Acute abdomen	49 (3.2)	0.4 (0.7)
Gastroenteritis	44 (2.9)	0.4 (0.8)
Chest pain or ACS	43 (2.8)	0.3 (0.7)
Psychological disorders	35 (2.3)	0.3 (0.6)
Respiratory diseases^*^	27 (1.8)	0.2 (0.5)
Fever	20 (1.3)	0.2 (0.4)
Eye diseases	15 (1.0)	0.1 (0.4)
Seizure	15 (1.0)	0.1 (0.4)
UTI	10 (0.7)	0.1 (0.3)
Acute stroke	10 (0.7)	0.1 (0.4)
Diabetes-related illness	8 (0.5)	0.1 (0.3)
Gastro-intestinal bleeding	7 (0.5)	0.1 (0.2)

SD: standard deviation; ACS: acute coronary syndrome; UTI: urinary tract infection

*Pulmonary diseases include pneumonia, asthma, and chronic obstructive pulmonary disease

Among the 702 non-trauma-related cases, the most common diagnoses were heat emergencies (16.1%, *n* = 244), including dizziness, cramps, stress, and heat stroke. Other diagnoses reported in more than 2% of cases included skin diseases, acute abdomen, gastroenteritis, chest pain or acute coronary syndrome, and psychological disorders.

To investigate the associated factors of common diseases in the Mazu pilgrimages, the environmental factors during the events were collected. The daily highest temperature was 28.8 (SD = 4.1) °C, and the daily relative humidity was 78.0 (SD = 9.3) g/m^3^. According to the GPS records of the pilgrimages, the daily walking distance was 35.3 (SD = 19.4) km. The multivariable analysis showed that the S-or-E-day variable was significantly associated with daily number of ED visits, trauma cases, head injuries, orthopaedic injuries, and heat emergencies (Table 4). Moreover, walking distance was significantly associated with daily number of ED visits and trauma cases, whereas the highest temperature was significantly associated with number of heat emergencies.

**Table 4:** Multivariable linear regression models on outcomes. (*n* = 124)

Outcome	Adjusted *R*-squared	S-or-E-day		WD (km)		HT (°C)		RH (%)	
		(β, 95% CI)	(Exp(β), %)	(β, 95% CI)	(Exp(β), %)	(β, 95% CI)	(Exp(β), %)	(β, 95% CI)	(Exp(β), %)
Number of ED visits	0.204	0.381 (0.221, 0.542)	1.464, + 46.4%	0.004 (0.0005, 0.007)	1.004, + 0.4%	-0.003 (-0.02, 0.013)	0.997, -0.3%	-0.012 (-0.019, -0.004)	0.988, -1.2%
Trauma	0.215	0.409 (0.249, 0.569)	1.506, + 50.6%	0.004 (0.0003, 0.007)	1.004, + 0.4%	-0.008 (-0.025, 0.008)	0.992, -0.8%	-0.011 (-0.018, -0.004)	0.989, -1.1%
Head injuries	0.143	0.258 (0.145, 0.37)	1.295, + 29.5%	0.001 (-0.001, 0.004)	1.001, + 0.1%	-0.005 (-0.017, 0.007)	0.995, -0.5%	-0.004 (-0.009, 0.0009)	0.996, -0.4%
Orthopaedic injuries	0.046	0.125 (0.039, 0.21)	1.133, + 13.3%	0.001 (-0.001, 0.003)	1.001, + 0.1%	-0.0009 (-0.01, 0.008)	0.999, -0.09%	-0.001 (-0.005, 0.002)	0.999, -0.1%
Heat emergencies	0.086	0.183 (0.059, 0.308)	1.201, + 20.1%	-0.00005 (-0.003, 0.002)	0.99995, ~ 0%	0.013 (0.0005, 0.026)	1.013, + 1.3%	-0.004 (-0.01, 0.002)	0.996, -0.4%

S-or-E-day: the start or end day of pilgrimage; WD: walking distance; HT: highest temperature; RH: relative humidity; CI: confidence interval

(β, 95% CI) = estimated effect and its 95% CI

(Exp(β), %) = exponentiated coefficient and percent change relative to the baseline

In the multivariable regression models, the S-or-E-day variable was the primary independent variable of interest. After log-transformation of the outcomes, each additional day closer to the end of the pilgrimage was associated with a 46.4% increase in the daily number of ED visits (exp(β) = 1.464, 95% CI: 1.25–1.68). This means that, holding other variables constant, the expected number of ED visits increases by approximately 46% for each day closer to the pilgrimage’s conclusion. Similar interpretations apply to trauma cases (+ 50.6%) and other subgroups, as detailed in Table 4.

## Discussion

Historically, mass religious gatherings are significant cultural phenomena that have contributed substantially to the local economy. However, they also pose potential threats and place significant stress on local healthcare systems, especially for events involving a large number of participants, such as Mazu pilgrimages in Taiwan. Our study revealed a significant increase in ED visits at the start or end of the pilgrimages, when the highest stress was placed on the healthcare system. Furthermore, the health impacts extended beyond EDs to the emergency medical services (EMS), which had to respond to the increased demand for ambulance transportation. Therefore, it is critical to effectively manage both prehospital EMS and emergency services in EDs to bridge potential treatment gaps. Our study provides a comprehensive view of how moving mass religious gatherings affect the healthcare system.

Trauma accounted for half of the health issues observed during these events, although most did not require hospital admission. Additionally, there was a significant association between daily walking distance and number of trauma cases. This suggests that, when the pilgrimage schedule includes extended walking distances, the healthcare system should be prepared for a potential increase in demand for emergency care. While soft tissue injuries were the most common type of trauma observed, head injuries were the second most common type. This may have resulted from overcrowding and the dynamics during the pilgrimage. Unlike previous studies on Hajj [11], which did not highlight head injuries as a major concern, our findings indicate that evaluation of head injuries should be emphasised in similar pilgrimage events in the future.

We also found that the start or end day of pilgrimage were independent predictors of all trauma-related conditions, particularly orthopaedic and head injuries. Mazu pilgrimages are characterised by periods of mass gatherings in confined spaces around temples during the opening and closing ceremonies. These conditions likely contribute to a substantial increase in the risk of trauma [9]. Moreover, the potential for mass casualty incidents exists, similar to previous events such as the Mina Stampede in 2015 [10, 12] and the Seoul Halloween crowd crush in 2022 [13]. These examples highlight the importance of implementing effective crowd management and safety measures to mitigate the risk of severe trauma and prevent similar incidents in future pilgrimages [14]. 

For non-traumatic conditions, communicable diseases have traditionally been considered significant concerns [1, 2, 7]. However, we reported only few cases of communicable diseases, with gastroenteritis being the primary concern. In contrast, heat emergencies of varying degrees were the most common non-trauma-related diagnosis in our study. This finding aligns with those of recent research emphasising the health impacts of climate change during Hajj [5, 6, 8]. Although the daily temperatures observed during Hajj are generally higher than those recorded in our study, we found a positive association between the daily maximum temperature and the incidence of heat emergencies, which aligns with findings from the cold cycle of Hajj from 1996 to 2014 [8]. These results underscore the importance of considering environmental factors, such as temperature, especially within 20–30 °C, when preparing for mass gatherings, to prevent heat-related health issues.

Because mass religious gatherings are usually scheduled and predictable, using previous data for preparedness before the events is critical for mitigating potential health impacts. However, management strategies are sometimes in conflict with religious practices. For instance, the route of the Baishatun Mazu pilgrimage is not pre-scheduled and is decided randomly each day, posing even greater challenges to the healthcare system. With advancements in artificial intelligence, health authorities and emergency managers can enhance their prediction and warning capabilities based on current data. In recent years, pilgrimage organisers have used real-time global positioning systems to track and display the location of Mazu pilgrimages, which can help localise potential health demands and allocate related resources more effectively. Additionally, health authorities have begun to analyse real-time data, including environmental factors, to assess medical needs. This enables the optimal distribution of mobile medical units and first-aid teams that can move alongside the pilgrims, ensuring prompt responses to emergencies.

## Limitations

This study has some limitations. First, our findings may not be generalisable to other events owing to the varying characteristics of mass-gathering events. In addition, the sample size was small, and data were collected over a 7-year period from only two pilgrimages. Nevertheless, given the infrequency of such large-scale events worldwide, our data could serve as a reference for planning similar events, whether they involve mass gatherings in confined spaces or moving tours with large numbers of participants.

Second, due to the lack of precise data on the actual number of participants, we were unable to calculate the incidence rates and could only report the absolute number of occurrences. This limitation makes it challenging to provide conclusive evidence on the relative risk associated with the activities on each pilgrimage day. However, we attempted to quantify health needs in terms of both number and magnitude to estimate potential health impacts and improve preparedness. It is important to note that the magnitude of health needs may vary, given the large number of participants.

Third, we only collected patient data reported by the receiving EDs. Patients treated by mobile medical units and first aid teams were not included, which may have led to an underestimation of the total number of patients. However, these patients typically present with mild injuries or illnesses that do not significantly limit their medical resources. Thus, their exclusion may not substantially affect the preparedness strategies for EDs and hospitals. Further studies should include data from all healthcare providers involved in such events to provide a more comprehensive view of healthcare demand and resource allocation.

## Conclusions

This study offers a comprehensive analysis of the health impacts associated with a long-distance, multi-day mass religious event. We identified key factors—particularly trauma and heat-related emergencies—associated with increased emergency department utilization. These findings can help health authorities better anticipate medical needs by monitoring walking distance, temperature, and other critical variables in advance, allowing for timely interventions. Further validation and real-world application of these results are warranted.

Future research may explore the effectiveness of specific interventions, such as enhanced management during high-risk time periods, integration of geographic information systems, and early warning mechanisms. Incorporating such tools may enable more precise and responsive health strategies, ultimately improving the cost-effectiveness of emergency preparedness and response for large-scale cultural events.

## Electronic supplementary material

Below is the link to the electronic supplementary material.

Supplementary Material 1

Supplementary Material 2

## Data Availability

The data that support the findings of this study are available from the authors but restrictions apply to the availability of these data, which were used under license from data from the Ministry of Health and Welfare (Taiwan) for the current study, and so are not publicly available. Data are, however, available from the authors upon reasonable request and with permission from the Ministry of Health and Welfare (Taiwan).

## Abbreviations

ED: Emergency department
EMRMS: Emergency Medical Resources Management System
TTAS: Taiwan Triage and Acuity Scale
SD: Standard deviation
EMS: Emergency medical services
