# Supplementary material for: Assessing health risks and preparedness strategies in mass-gathering religious events: a retrospective observational study
Source: BMC Emerg Med. 2025 Jul 21;25:132. doi: 10.1186/s12873-025-01293-x (PMC12281752; doi:10.1186/s12873-025-01293-x)
Supplement: Supplementary file 1 — Supplementary Material 1 [file 12873_2025_1293_MOESM1_ESM.docx]

# Supplementary Table 1. Taiwan Triage and Acuity Scale (TTAS)

| TTAS Level | Category | Estimated Wait Time | Examples |
| --- | --- | --- | --- |
| I | Resuscitation | Immediate  (0 minutes) | - Cardiac or respiratory arrest - Cyanosis of limbs/lips - Body temperature >41°C or <32°C - Unconsciousness or altered consciousness - Continuous seizure with unconsciousness |
| II | Emergency | 10 minutes | - Acute change in consciousness (slowed speech and movement but still able to communicate) - Persistent chest tightness or chest pain with cold sweating - Hypoglycemia (<40 mg/dL) - Large amounts of hematochezia, melena, or hematemesis - Severe bleeding from trauma, with uncontrolled hemorrhage from the head, neck, trunk, or pelvis - Gunshot wounds; blunt or penetrating injuries to the head, neck, or trunk; open wounds with suspected fractures - Fall from height, ejection from a vehicle in a traffic accident, or loss of consciousness after head trauma - Neurological abnormalities (motor or sensory changes) following trauma or chemical exposure - Suspected drug allergy causing difficulty breathing - Stings or bites leading to respiratory distress or altered consciousness |
| III | Urgent | 30 minutes | - Obvious shortness of breath while walking - Abdominal pain with missed menstrual period - Uncontrollable diarrhea or vomiting - Swollen and deformed limb after trauma, suspected fracture/dislocation - Coffee-ground vomitus or black stool - Hypertension (systolic >200 mmHg or diastolic >110 mmHg) without any symptoms - Consciousness regained after seizure - Widespread rash or blisters - Inhalation of toxic gas or other gases without signs of respiratory distress |
| IV | Less Urgent | 60 minutes | - Localized cellulitis - Painful urination without fever - Acute cough without fever - Fever without other discomfort - Recurrent pain or dizziness - Persistent hiccups - Poor appetite or anorexia with stable vital signs |
| V | Non-Urgent | Within 120 minutes | - Habitual constipation - Chronic nausea, vomiting, or hiccups - Joint swelling - Minor abrasions, bruises, or soft tissue injuries - Insect or animal bites/stings without fever or significant discomfort - Allergy or nasal congestion - Chronic insomnia - Mild diarrhea without signs of dehydration |
