# Supplementary material for: Assessing health risks and preparedness strategies in mass-gathering religious events: a retrospective observational study
Source: BMC Emerg Med. 2025 Jul 21;25:132. doi: 10.1186/s12873-025-01293-x (PMC12281752; doi:10.1186/s12873-025-01293-x)
Supplement: Supplementary file 2 — Supplementary Material 2 [file 12873_2025_1293_MOESM2_ESM.docx]

Supplementary Table 2. Environmental factors during the Dajia Mazu and Baishatun Mazu pilgrimages between 2018 and 2024

| **Environmental factors** | Median | Mean (SD) | IQR | | Minimum | | Maximum | |  |
| --- | --- | --- | --- | --- | --- | --- | --- | --- | --- |
| Highest temperature | 29.2 | 28.8 (4.1) | | 5.3 | | 16.6 | | 36.9 | |
| Relative humidity | 78.0 | 78.0 (9.3) | | 10.5 | | 48.0 | | 99.0 | |
| Walking distance | 33.4 | 35.3 (19.4) | | 21.8 | | 0.0 | | 108.8 | |
